# Acute effect of exercise on appetite‐related factors in males with obesity: A pilot study

**DOI:** 10.14814/phy2.70167

**Published:** 2024-12-25

**Authors:** Sogand Asri, Farhad Rahmani‐nia, Payam Saidie, Timothy J. Fairchild, Shahin Khodabandeh

**Affiliations:** ^1^ Department of Exercise Physiology University of Guilan Guilan Iran; ^2^ Centre for Molecular Medicine and Innovative Therapeutics Health Futures Institute, Murdoch University Murdoch Western Australia Australia

**Keywords:** appetite perceptions, appetite‐regulation, IL‐6, IL‐7, irisin, NPY

## Abstract

To investigate the role of appetite‐related factors, including interleukin 6 (IL‐6), irisin, interleukin 7 (IL‐7), neuropeptide Y (NPY), and leptin, on appetite perception in males with obesity. Eleven males (BMI 35.3 ± 4.2 kg/m^2^, V̇O_2peak_ 29 ± 3.1 mL/kg/min) participated in two experimental trials (MICE: 60 min of cycling at 60% of V̇O_2peak_; CTRL: 60 min of quiet resting) using a crossover design. Appetite parameters, including IL‐6, IL‐7, irisin, and leptin, were measured. Additionally, appetite perception was assessed. IL‐6 concentration increased significantly immediately post‐exercise (95% CI: [2.207–12.192] pg/mL, *p* = 0.007) and remained elevated 1 hour post‐exercise (95% CI: [2.326–11.855] pg/mL, *p* = 0.006) compared to CTRL. Irisin also rose significantly immediately post‐exercise (95% CI: [0.084–3.061] ng/mL, *p* = 0.039). NPY decreased significantly 1 h post‐exercise (95% CI: [(−20.601) – (−1.380)] ng/L, *p* = 0.027). No significant differences were observed for IL‐7 (*p* = 0.748, $\eta_{p}^{2}$ = 0.077) and leptin (*p* = 0.285, $\eta_{p}^{2}=0.061$). Appetite perceptions were suppressed immediately post‐exercise (95% CI: [3.407–19.547] mm, *p* = 0.008) compared to CTRL. Sixty minutes of MICE increased IL‐6 and irisin concentrations while suppressed NPY and appetite perceptions in males with obesity.

## 1 INTRODUCTION

Obesity is one of the main challenges faced by the majority of countries in the contemporary period (Janssen et al., 2020). While the complex etiology of obesity involves an interplay of diverse factors, it is widely acknowledged that eating behavior is a significant contributing factor (Bray, 2004). Appetite regulation is affected by several psychological, environmental, and physiological factors that are centrally controlled by the brain (Watts et al., 2022). Strategies aimed at addressing obesity‐related eating habits are essential in combating the obesity epidemic. Developing effective interventions requires a thorough understanding of the underlying mechanisms. Various tissues, organs, hormones, and neural circuits exert potential influences within the appetite control system, which may be sensitive to changes induced by exercise (Thackray & Stensel, 2023).

In recent decades, most studies investigating the interaction between appetite and exercise have focused on gastrointestinal hormones (Dorling et al., 2018). However, recent evidence highlights the importance of certain myokines, adipokines, and cytokines in appetite regulation (Grannell et al., 2022). In response to skeletal muscle contraction during exercise, multiple muscle‐derived peptides are released that may impact appetite. For instance, interleukin‐6 (IL‐6), an inflammatory cytokine released from skeletal muscle in an intensity‐dependent manner, is hypothesized to regulate appetite following acute exercise (Islam et al., 2017). Similarly, interleukin‐7 (IL‐7) which is released from skeletal muscle in response to contraction, has been shown to impact appetite by binding its receptors located on the arcuate nucleus in mice, a brain region involved in appetite regulation (Macia et al., 2010). Irisin is another exercise myokine that has co‐localization receptors with Neuropeptide Y (NPY) in the paraventricular nucleus and is associated with appetite suppression (Huh et al., 2012). Neuropeptide Y is considered the most important orexigenic neuropeptide in the central nervous system. However, there is limited research indicating whether systemic NPY may also affect appetite (Tyszkiewicz‐Nwafor et al., 2021). Leptin is best known as an adipokine, but is also released from skeletal muscle and is involved in energy regulation, with increasing concentrations leading to satiety (Ahima & Flier, 2000).

Evidence indicates that the response of appetite hormones in people with obesity differ to those in lean individuals (Aukan et al., 2023). Moderate continuous aerobic exercise (50%–70% V̇O_2peak_) can induce the feeling of satiety in lean people commensurate with fluctuation in gastrointestinal (GI) hormones, while the relationship of these hormones are less consistent in people living with obesity (Dorling et al., 2018). Additionally, only a few studies have focused on other appetite‐related factors such as the myokines and cytokines in response to exercise in individuals with obesity. Bornath et al. reported 60 min of aerobic exercise at 65% V̇O_2max_ increased the concentration of IL‐6 in both lean and obese participants, but did not appear to influence subjective appetite (Bornath et al., 2023). Additionally, there were contradictory results for irisin. Herrera et al. reported that Irisin concentration did not alter after 45 min aerobic exercise at 65% HR_peak_ (Archundia‐Herrera et al., 2017) while LeBlanc et al. reported that MICE at 60% of heart rate reserve for 45 min increased irisin concentration (Blizzard LeBlanc et al., 2017). There is a lack of studies investigating the effects of exercise on IL‐7. Only one study has reported that IL‐7 levels increased after 90 min of playing football, but this study did not focus on the appetite‐related aspects of IL‐7 (Andersson et al., 2010).

Despite the majority of studies reporting no effects of moderate aerobic exercise on GI hormones, subjective appetite suppression has been observed in individuals with overweight or obesity. This study is, to our knowledge, the first to explore the impact of acute moderate aerobic exercise on IL‐7 in individuals with obesity. In the current study, we aim to elucidate the underlying reasons for immediate and subsequent appetite suppression following acute aerobic exercise in individuals with obesity. We hypothesize that fluctuations in appetite‐related factors may induce appetite suppression following the MICE.

## 2 METHOD

The data presented in this study was part of a larger project (Khodabandeh et al., 2024) which was approved by the Sport Sciences Research Institute of Iran's Ethics Advisory Committee. All participants provided written informed consent prior to their inclusion in the study. Following that, eleven males with obesity (body weight: 109.8 ± 16.4 kg; age: 22 ± 2 y; body mass index, BMI: 35.3 ± 4.2 kg/m^2^; waist circumference: 119.6 ± 7.2 cm; body fat percentage: 36.5 ± 2.5%; V̇O_2peak_: 29 ± 3.1 mL⋅kg^−1^⋅min^−1^) were recruited to participate. The following requirements were met by all participants: non‐smoking, body mass stability in the previous 3 months (±2 kg), metabolically healthy, using no medications, living an inactive lifestyle based on the International Physical Activity Questionnaire (Craig et al., 2003), with body fat between 32 and 45 percent, BMI >30 kg/m^2^, and waist circumference >102 cm.

### 2.1 Preliminary session

To be eligible participants, individuals were instructed not to engage in physical activity on the day before the measurement session. They were also required to arrive after 12 h of fasting and to abstain from drinking any liquids, including water, for 8 h to ensure accurate bioelectrical impedance measurements. To determine metabolic health, blood samples were collected to measure high‐density lipoprotein (HDL), low‐density lipoprotein (LDL), triglycerides (TG), and total cholesterol. Additionally, height was measured using an electronic measuring station (Seca, Hamburg, Germany) as part of standard anthropometric assessment. Body fat percentage was assessed using an InBody 270 device (InBody, Seoul, South Korea). Waist circumference was measured at the narrowest point of the torso, between the lower rib margin and the iliac crest. After these measurements, participants were provided with a standard breakfast and given 1 h for digestion. During this time, participants completed a set of questionnaires, including those on health status and habitual physical activity using the International Physical Activity Questionnaire. Participants underwent a peak oxygen uptake test on a cycle ergometer (Monark LC4, Monark Exercise AB, Vansbro, Sweden). The test began with a 5‐min warm‐up at a resistance of 50 W. After the warm‐up, the workload increased by 20 W every 2 min until the participants reached exhaustion, maintaining a pedaling rate of 60–70 rpm. During the test, oxygen consumption and carbon dioxide production were measured using an online breath‐by‐breath gas analysis system (Metalyzer 3B, Cortex, Biophysik, Germany). Additionally, heart rate was monitored using a Polar H10 (Polar Electro Oy). To ensure participants reached their peak oxygen uptake (V̇O_2peak_), they needed to meet at least two of the following criteria: heart rate within ten beats per minute of the age‐predicted maximum, a respiratory exchange ratio (RER) of 1.1 or higher, and a rating of perceived exertion (RPE) of 19 or higher.

### 2.2 Experimental procedure

Participants completed two 4‐h trials in a randomized crossover design: one trial included exercise, and the other did not. These trials were separated by a minimum of 7 days. Participants were asked to not consume alcohol and caffeine or take part in any exercise 48 h before the tests. Participants were instructed to replicate their food and beverage intake as closely as possible for the 24 h prior to each session, using a food diary to maintain consistency. The night before each experimental trial, they were provided with a standardized dinner of macaroni and beef, designed to supply 40% of their daily energy requirement (408 ± 4905 kJ) according to the Mifflin‐St Jeor equation, with a macronutrient composition of 50% carbohydrate, 38% fat, and 12% protein (Mifflin et al., 1990). Participants were also instructed to refrain from consuming anything except water after 10 pm and to sleep for a minimum of 7 h. After they arrived at the lab at 8 am for the experimental sessions, participants were provided with a standardized breakfast to consume in 15 min. The standardized breakfast—consisting of white bread, strawberry jam, margarine, banana, and orange juice—provided 30% of participants' estimated daily energy needs, calculated using the Mifflin equation. The average energy content of the breakfast was 3678 kJ, with macronutrient distribution of 71% carbohydrates, 19% fat, and 10% protein. Energy and macronutrient values were determined based on manufacturer‐provided data. One hour rest was considered for digestion before beginning the exercise session. At this stage, participants either rested for 60 min or exercised for 60 min at a moderate intensity (60% V̇O_2peak_) on a cycle ergometer. During the exercise session, an online breath‐by‐breath gas analysis system was utilized to measure energy expenditure and monitor heart rate, consistent with the methods described earlier.

### 2.3 Subjective appetite perceptions

**FIGURE 1:**
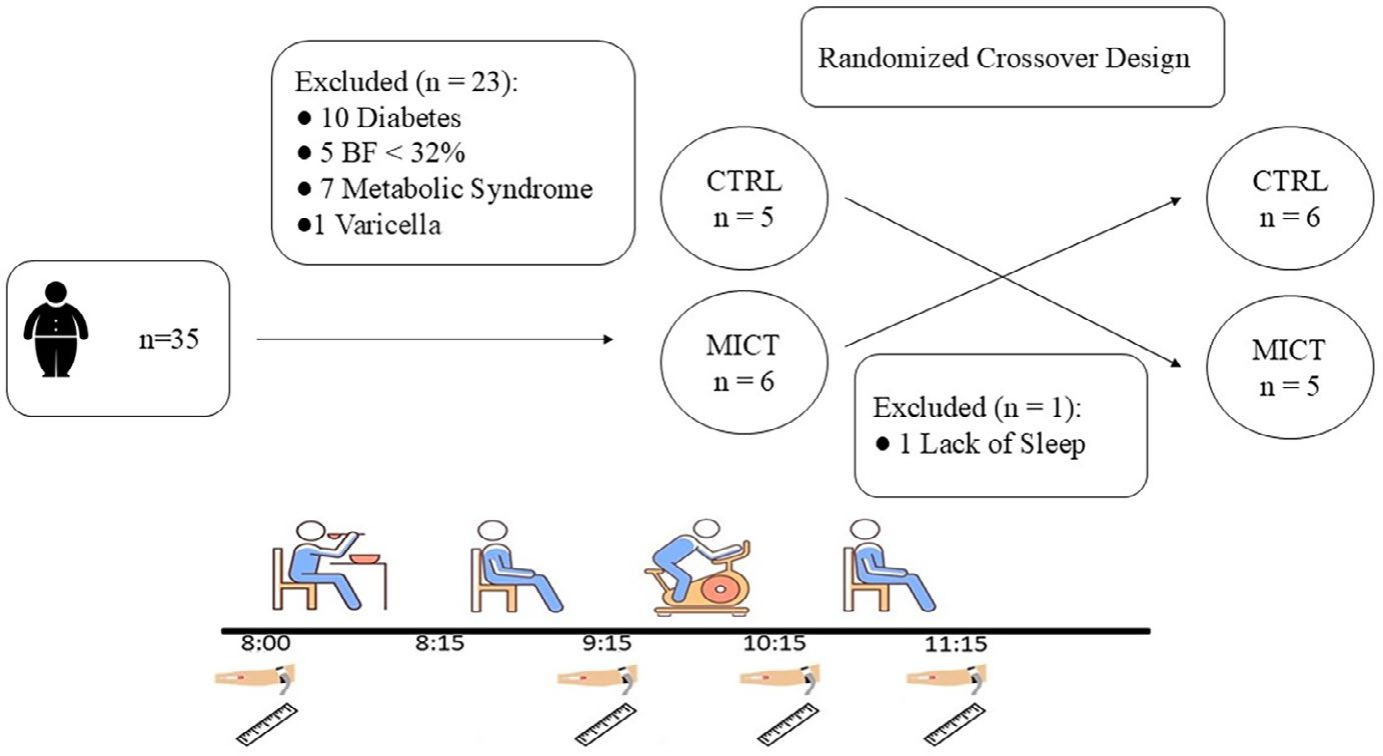
Flowchart illustrating participant selection and study design. Blood sampling times are marked with a hand icon, and appetite perception is represented by a ruler icon.

Appetite perceptions were assessed before breakfast, 30 min after breakfast, and at 30‐min intervals until 11:15 am using a 100 mm visual analog scale. The questions addressed hunger, satiation, fullness, and desire to eat. The overall appetite rating was calculated by averaging the four appetite ratings, with the scores for satisfaction and fullness being reverse‐scored (Flint et al., 2000) (Figure 1).

### 2.4 Blood sampling

All samples were collected at 0 minutes (fasted), 75 min (post‐prandial, pre‐exercise), 135 min (immediately post‐exercise), and 195 min (60 min post‐exercise) of each session via venipuncture from different points of the antecubital vein while participants remained seated. Venous blood samples were drawn into EDTA vacuum tubes (Xinle, China) and centrifuged at 1750 ***g*** for 10 min at 4°C. The resulting supernatants were partitioned into storage tubes and preserved at −80°C. These aliquots were later analyzed in batches upon study completion to minimize assay variability. IL‐6 (ZB‐10090C‐H9648), irisin (ZB‐13253C‐H9648), IL‐7 (ZB‐11948C‐H9648), NPY (ZB‐11258C‐H9648), and leptin (ZB‐11559C‐H9648) were measured according to the ZellBio ELISA kit instructions (Human ELISA Kits, ZellBio GmbH, Ulm, Germany). The intra‐assay coefficient of variation (CV) for IL‐6, irisin, IL‐7, NPY, and leptin was less than 10%, and the inter‐assay CV was less than 12%. (CV(%) = (SD/MEAN)).

### 2.5 Sample size calculation

A sample size estimate was conducted using G*Power 3.1. With an effect size of *f* = 0.5, based on the session × time difference (partial eta squared = 0.2) observed in IL‐6 in a similar study design (Bornath et al., 2023), and with alpha set at 0.05 and power at 0.80, it was determined that a minimum of 11 participants would be required to detect significant differences (Stubbs et al., 2000).

### 2.6 Statistical analysis

Data were analyzed using SPSS software version 29 (IBM Corporation, Armonk, NY, USA) for Windows. A repeated‐measures two‐factor ANOVA assessed variations between trials over time concerning appetite and related parameters. When significant main and interaction effects were identified, post hoc analysis was conducted using the Holm‐Bonferroni correction to address multiple comparisons. An independent *t*‐test was used to compare area under the curve (AUC) values, which were calculated using the trapezoidal approximation method. Statistical significance was accepted at *p* < 0.05. The 95% confidence intervals (CI) were calculated for mean absolute pairwise differences between experimental trials and sessions. Effect sizes are provided to complement the results, including partial eta squared ($\eta_{p}^{2}$; small 0.01, medium 0.06, large 0.14) for main effects and interactions.

## 3 RESULTS

### 3.1 Il‐6

**FIGURE 2:**
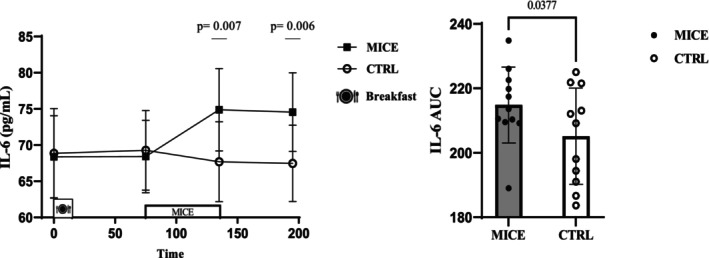
IL‐6 concentrations across all time points during each experimental session. AUC for IL‐6 across time points during each experimental session.

There was a time × session interaction for IL‐6 (*p* = 0.005, $\eta_{p}^{2}=0.275$) while IL‐6 increased significantly immediately after exercise (95% CI: [2.207–12.192] pg/mL, *p* = 0.007) and remained elevated 1 h post‐exercise (95% CI: [2.326–11.855] pg/mL, *p* = 0.006) compared to CTRL. The concentrations of IL‐6 were also increased immediately post‐exercise compared to pre‐exercise (95% CI: [1.507–11.419] pg/mL, *p* = 0.006) and fasting (95% CI: [0.600–12.399] pg/mL, *p* = 0.025) and were also higher 1 h post‐exercise compared to pre‐exercise (95% CI: [0.793–11.461] pg/mL, *p* = 0.019) and fasting (95% CI: [0.159–12.168] pg/mL, *p* = 0.042). Also, the AUC of IL‐6 was significantly higher in the MICE session compared to the CTRL session (*p* = 0.037) (Figure 2), and this was due to the exercise‐related response (Figure S1) Table 1.

**TABLE 1:** Response to exercise.

Variables	Mean ± SD
Heart rate (beats min ^−1^)	142 ± 13
Workload (Watts)	80 ± 30
Energy expenditure (kJ)	2724 ± 585
Fat oxidation(g)	26.18 ± 7
Carbohydrate (g) oxidation	73.77 ± 31

### 3.2 Irisin

**FIGURE 3:**
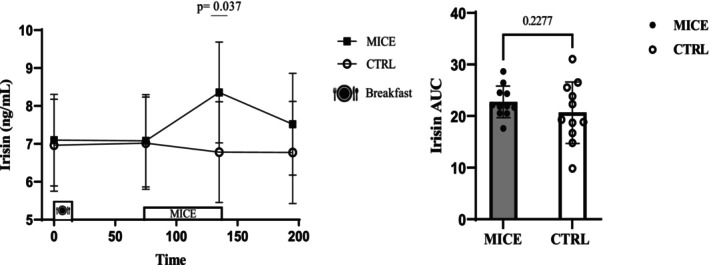
Irisin concentrations across all time points during each experimental session. AUC for irisin across time points during each experimental session.

There was a time × session interaction for irisin (*p* = 0.012, $\eta_{p}^{2}=0.195$) while irisin increased significantly immediately after exercise (95% CI: [0.084–3.061] ng/mL, *p* = 0.039) compared to CTRL. The concentrations of irisin were also increased immediately post‐exercise compared to pre‐exercise (95% CI: [0.446–2.099] ng/mL, *p* = 0.001) and fasting (95% CI: [0.010–1.661] ng/mL, *p* = 0.046). However, there was no difference for irisin AUC (*p* = 0.227) (Figure 3), even when separated into the irisin response to meals or exercise (Figure S2).

### 3.3 Il‐7

**FIGURE 4:**
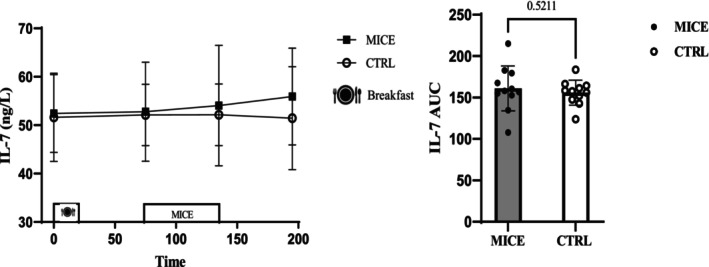
IL‐7 concentrations across all time points during each experimental session. AUC for IL‐7 across time points during each experimental session.

There was no interaction (Time × Session) (*p* = 0.748, $\eta_{p}^{2}=0.077$) or main effect of session (*p* = 0.524, $\eta_{p}^{2}=0.011$) for IL‐7, and there was not a main effect of time (*p* = 0.826, $\eta_{p}^{2}=0.343$). Additionally, no differences were observed in IL‐7 AUC (*p* = 0.521) (Figure 4), even when analyzed separately for responses to meals or exercise (Figure S3).

### 3.4 NPY

**FIGURE 5:**
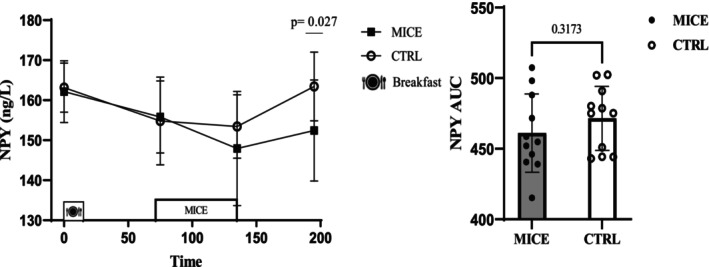
NPY concentrations across all time points during each experimental session. AUC for NPY across time points during each experimental session. * donates significant changes.

There was a time × session interaction for NPY (*p* = 0.037, $\eta_{p}^{2}=0.142$). while NPY concentration was significantly lower in 1 h post‐exercise (95% CI: [(−20.601) – (−1.380)] ng/L, *p* = 0.027) compared to CTRL. The NPY concentration was significantly reduced immediately post‐exercise (95% CI: [(−22.646) – (−5.808)] ng/L, *p* < 0.001) and 1 h post‐exercise (95% CI: [(−18.443) – (−0.993)] ng/L, *p* = 0.024) compared to fasting in the exercise session. However, there was no difference for NPY AUC (*p* = 0.317) (Figure 3), even when separated into the NPY response to meals or exercise (Figure S2 and Figure 5).

### 3.5 Leptin

**FIGURE 6:**
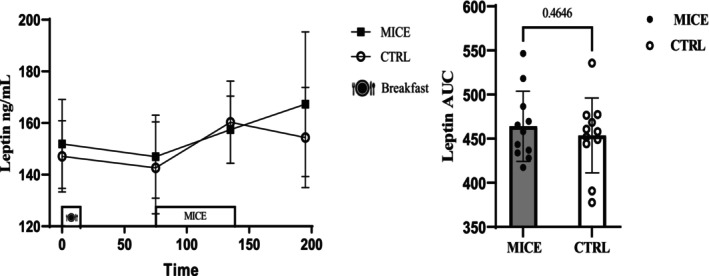
Leptin concentrations across all time points during each experimental session. AUC for leptin across time points during each experimental session.

There was no interaction (Time × Session) (*p* = 0.285, $\eta_{p}^{2}=0.061$) or main effect of session (*p* = 0.433, $\eta_{p}^{2}=0.031$) for leptin, but there was a main effect of time (*p* = 0.002, $\eta_{p}^{2}=0.265$). Additionally, no differences were observed in leptin AUC (*p* = 0.464) (Figure 6), even when analyzed separately for responses to meals or exercise (Figure S3).

### 3.6 Appetite perceptions

**FIGURE 7:**
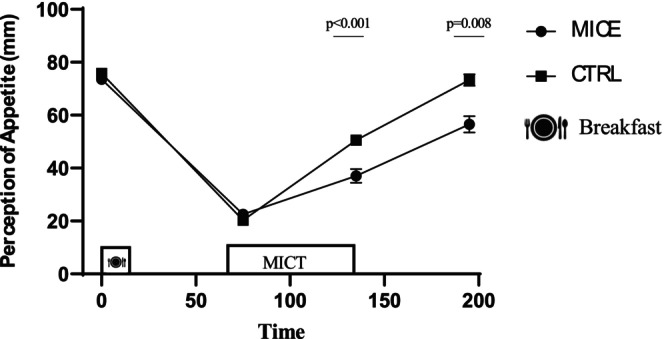
Perception of appetite across all time points during each experimental session. * Denotes significantly different from control condition.

There was a time × session interaction for appetite perception (*p* < 0.001, $\eta_{p}^{2}=0.322$) while appetite perception rates were significantly lower immediately post‐exercise (95% CI: [3.407–19.547] mm, *p* = 0.008) and 1 h post‐exercise (95% CI: [6.252–22.385] mm, *p* = 0.001) compared to CTRL (Figure 7).

## 4 DISCUSSION

To our knowledge, this is the first study that explored the role of IL‐7 following acute moderate aerobic exercise in sedentary people with obesity. The key findings of this study include (I) elevation of IL‐6 and irisin after exercise between sessions, (II) reduction of NPY 1 h post‐exercise, (III) IL‐7 and leptin did not significantly alter by exercise, and (IV) subjective appetite was suppressed following acute aerobic exercise between sessions.

In line with previous studies (Bornath et al., 2023), IL‐6 increased significantly immediately post‐exercise and remained elevated 1 h post‐exercise between sessions. Additionally, the AUC of IL‐6 was significantly higher in the MICE session compared to the CTRL session. Andarianto et al. indicated that 40 min of MICE at 60%–70% of HR_max_ increases the concentration of IL‐6 in women with obesity (Andarianto et al., 2022). This increase in IL‐6 appears to depend on the number of muscles recruited during exercise and the duration of the exercise (Nash et al., 2023). The depletion of glycogen resources can stimulate the release of IL‐6 (Gleeson, 2000), with evidence indicating that slow‐twitch muscle fibers are the main source of this release (Plomgaard et al., 2005). Consequently, MICE recruited these types of muscles, which may explain the increase in IL‐6 observed. It has been proposed that IL‐6 may impact appetite regulation by stimulating GLP‐1 and PYY (Ellingsgaard et al., 2020). However, Bornath et al. reported that an increase in IL‐6 after 60 min of MICE at 65% V̇O_2max_ did not affect the concentration of active GLP‐1 in people with obesity (Bornath et al., 2023). Additionally, IL‐6 can influence appetite perception by exerting its effects centrally within the hypothalamus. Research indicates that IL‐6 is released by the muscles into the bloodstream, allowing it to traverse the blood–brain barrier and impact appetite (Banks et al., 1994). Nonetheless, administering a peripheral injection with a concentration of IL‐6 that was four times higher resulted in a significant decrease in food consumption (Timper et al., 2017). There is evidence showing that exercise can increase the level of IL‐6 up to 100‐fold (Steensberg et al., 2000). Thus, it seems that IL‐6 can play a crucial role in exercise‐induced anorexia.

The role of IL‐7 in appetite regulation has come to light in recent rodent studies, demonstrating that endogenous IL‐7 reduces energy intake by stimulating POMC (Macia et al., 2010). However, the precise underlying mechanism remains unclear. Only one study has explored the impact of football exercise on IL‐7, reporting an elevation in its levels (Andersson et al., 2010). In our investigation, we observed that moderate aerobic exercise did not lead to significant alterations in IL‐7 levels. This could potentially be attributed to the exercise intensity, which may not have been sufficient to increase IL‐7 levels, despite previous reports suggesting higher IL‐7 levels in individuals with obesity (Germain et al., 2016). Based on our findings, it does not seem that IL‐7 can affect appetite suppression in response to exercise in males with obesity.

Our findings indicate an approximately 20% increase in irisin levels, which aligns with previous research showing an increase in irisin after 45 min of exercise at 60% of heart rate reserve in people with obesity and overweight (Blizzard LeBlanc et al., 2017). However, Archundia‐Herrera et al. did not observe a significant effect of MICE at 65% HR_peak_ for 40 min on irisin levels in women with obesity (Archundia‐Herrera et al., 2017). Peroxisome proliferator‐activated receptor γ coactivator 1α (PGC‐1α), produced by muscle activity during exercise, stimulates the expression of FNDC5, the gene encoding irisin (Huh et al., 2012). Adipocytes also release irisin into the bloodstream (Moreno‐Navarrete et al., 2013). Furthermore, studies have shown that injecting irisin into the hypothalamus reduces energy intake. This reduction is associated with a significant decrease in orexin‐A and an increase in pro‐opiomelanocortin (POMC) expression. Evidence indicates higher irisin concentrations in people with obesity, a phenomenon termed “irisin resistance” (Arias‐Loste et al., 2014). The duration of exercise appears to be a crucial factor in elevating irisin levels in response to physical activity (Bernal Rivas et al., 2022).

The concentration of leptin did not significantly change in response to MICE in the current study, aligning with the findings of Bornath et al., who also reported no alteration in leptin concentration due to MICE (Bornath et al., 2023). However, the reduction of leptin was reported after 60 min of MICE at 65% V̇O_2max_ in people with obesity and overweight (Tiryaki‐Sonmez et al., 2013). A recent study indicated that fasting prior to aerobic exercise can reduce leptin concentration (Fontana et al., 2023). Leptin is a well‐established hormone that plays a crucial role in both chronic and acute regulation of appetite and energy intake. Both central and peripheral administration of leptin has been shown to increase the mRNA expression of POMC/CART and decrease the expression of NPY/AgRP (Ahima & Flier, 2000).

NPY levels significantly reduced 1 h post‐exercise, a finding in contrast with previous studies reporting an increase in NPY levels following endurance exercise (25 km swim race in 6.9–10.5 h) in athletes (Karamouzis et al., 2002). NPY is one of the most important orexigenic neurotransmitters in the central nervous system and is also secreted by enteroendocrine cells in the intestine, sympathetic neurons of blood vessels, lymph tissue, and immune cells (Nergårdh et al., 2007). Although plasma NPY cannot cross the blood–brain barrier (Kastin & Akerstrom, 1999), some evidence suggests it can reduce energy intake. Notably, individuals with obesity tend to have higher concentrations of fasting plasma NPY. In line with previous research, plasma NPY may play a role in appetite suppression following MICE (Tyszkiewicz‐Nwafor et al., 2021). The duration of exercise appears to be a key factor influencing NPY concentration. However, further studies are necessary to compare the effects of different exercise intensities on NPY levels.

Studies have indicated that subjective appetite is transiently suppressed following acute moderate aerobic exercise performed at or above 60% V̇O_2peak_ (Dorling et al., 2018; Khodabandeh et al., 2024). Our findings support this, showing that moderate aerobic exercise can indeed suppress appetite perception in people with obesity. However, this result contrasts with a study that reported no significant appetite suppression after 60 min of MICE at 65% V̇O_2max_ (Bornath et al., 2023). The discrepancy between our findings and the study of Bornath et al. may be due the interaction between exercise and psychological factors such as stress, mood, and perceived exertion could also impact appetite regulation. Exercise‐induced changes in these factors might contribute to variations in appetite perception. Several mechanisms may explain the observed appetite suppression following moderate aerobic exercise. In the current study, the increase in IL‐6 and irisin, along with the reduction in NPY, may explain appetite suppression.

Many aspects of appetite regulation involve complex mechanisms that remain poorly understood. In recent years, the majority of studies have primarily focused on measuring gastrointestinal hormones, while other interconnected factors play a role in governing appetite including N‐lactoyl‐phenylalanine (Lac‐Phe), brain‐derived neurotrophic factor (BDNF), and asprosin (McCarthy et al., 2024). Additionally, there is a pressing need for studies that meticulously account for body composition in individuals with obesity to explore the relationship between myokines and appetite hormones in this specific population. Despite the valuable insights gained from this study, our findings need to be considered in light of several limitations associated with the study. A limitation of this study is the variability in the accuracy of commercially available kits for measuring irisin concentration, as recent research has questioned the reliability of these assays (Maak et al., 2021). Although the selected kit has been extensively used in prior studies and followed standardized protocols, it is acknowledged that inconsistencies in irisin quantification both between ELISA kits, as well as between ELISA kits and other methodologies (e.g., mass spectrometry) exist. Future studies may benefit from alternative methods or emerging technologies, once established in vivo, to confirm these results more robustly. Another limitation was our use of bioelectrical impedance to measure body fat, whereas subsequent research recommends employing DEXA to assess body composition, particularly visceral and ectopic fat.

## 5 CONCLUSION

Our findings show that IL‐6 and irisin increased after moderate aerobic exercise, which may contribute to suppressing appetite perceptions. However, IL‐7, NPY, and leptin did not significantly change as a result of the exercise. Despite the appetite suppression observed after MICE, it appears to be an effective approach for achieving energy balance in weight management.

## AUTHOR CONTRIBUTIONS

Conceptualization, F.R and Sh.Kh; methodology, T.F and Sh.Kh.; software, Sh.Kh.; validation, F.R and P.S; formal analysis, Sh.Kh, T.F; investigation, S.A and Sh Kh; data curation, Sh.Kh and S.A; writing original draft preparation, Sh.Kh and S.A; writing review and editing, F.R, P.S., and T.F; supervision, F.R.

## FUNDING INFORMATION

No funding information provided.

## CONFLICT OF INTEREST STATEMENT

The authors declare that they have no conflicts of interest relevant to this research project. There are no financial or personal relationships with other people or organizations that could inappropriately influence or bias the content of this manuscript.

## ETHICS STATEMENT

This study was approved by the Ethics Advisory Committee of the Sport Sciences Research Institute of Iran (IR.SSRC.REC.1402.116), and written informed consent was obtained from all participants prior to their involvement in the study.

## Supporting information


Figure S1.

## Data Availability

The datasets used and/or analyzed during the current study are available from the corresponding author upon reasonable request.
