# Supplementary material for: Acute effect of exercise on appetite‐related factors in males with obesity: A pilot study
Source: Physiol Rep. 2024 Dec 25;12(24):e70167. doi: 10.14814/phy2.70167 (PMC11669573; doi:10.14814/phy2.70167)
Supplement: Supplementary file 1 — Figure S1. [file PHY2-12-e70167-s001.docx]

**iAUC:**

**Figure S1 (A) IL-6 concentrations at all time points during each experimental session. (B) AUC for IL-6 in response to the meal (AUC for 0 min to 75 min). (C) AUC for IL-6 in response to the exercise (AUC for 75 min to 195 min).**

**Figure S2 (A) Irisin concentrations at all time points during each experimental session. (B) AUC for Irisin in response to the meal (AUC for 0 min to 75 min). (C) AUC for Irisin in response to the exercise (AUC for 75 min to 195 min).**

**Figure S3 (A) IL-7 concentrations at all time points during each experimental session. (B) AUC for IL-7 in response to the meal (AUC for 0 min to 75 min). (C) AUC for IL-7 in response to the exercise (AUC for 75 min to 195 min).**

**Figure S4 (A) Leptin concentrations at all time points during each experimental session. (B) AUC for Leptin in response to the meal (AUC for 0 min to 75 min). (C) AUC for Leptin in response to the exercise (AUC for 75 min to 195 min).**

**Figure S5 (A) NPY concentrations at all time points during each experimental session. (B) AUC for NPY in response to the meal (AUC for 0 min to 75 min). (C) AUC for NPY in response to the exercise (AUC for 75 min to 195 min).**
